# Vaccine Hesitancy and Rejection of a Vaccine for the Novel Coronavirus in the United States

**DOI:** 10.3389/fimmu.2021.558270

**Published:** 2021-06-14

**Authors:** Shu-Fang Shih, Abram L. Wagner, Nina B. Masters, Lisa A. Prosser, Yihan Lu, Brian J. Zikmund-Fisher

**Affiliations:** ^1^ Department of Health Administration, College of Health Professions, Virginia Commonwealth University, Richmond, VA, United States; ^2^ Department of Epidemiology, School of Public Health, University of Michigan, Ann Arbor, MI, United States; ^3^ Susan B. Meister Child Health Evaluation and Research Center, Department of Pediatrics and Communicable Diseases, University of Michigan Medical School, Ann Arbor, MI, United States; ^4^ Department of Epidemiology, Key Laboratory of Public Health Safety (Ministry of Education), Fudan University School of Public Health, Shanghai, China; ^5^ Department of Health Behavior & Health Education, School of Public Health, University of Michigan, Ann Arbor, MI, United States; ^6^ Department of Internal Medicine, Division of General Medicine, University of Michigan Medical School, Ann Arbor, MI, United States

**Keywords:** COVID-19, demography, disease outbreaks, vaccines, surveys and questionnaires

## Abstract

The arrival of the COVID-19 vaccine has been accompanied by increased discussion of vaccine hesitancy. However, it is unclear if there are shared patterns between general vaccine hesitancy and COVID-19 vaccine rejection, or if these are two different concepts. This study characterized rejection of a hypothetical COVID-19 vaccine, and compared patterns of association between general vaccine hesitancy and COVID-19 vaccine rejection. The survey was conducted online March 20-22, 2020. Participants answered questions on vaccine hesitancy and responded if they would accept the vaccine given different safety and effectiveness profiles. We assessed differences in COVID-19 rejection and general vaccine hesitancy through logistic regressions. Among 713 participants, 33.0% were vaccine hesitant, and 18.4% would reject a COVID-19 vaccine. Acceptance varied by effectiveness profile: 10.2% would reject a 95% effective COVID-19 vaccine, but 32.4% would reject a 50% effective vaccine. Those vaccine hesitant were significantly more likely to reject COVID-19 vaccination [odds ratio (OR): 5.56, 95% confidence interval (CI): 3.39, 9.11]. In multivariable logistic regression models, there were similar patterns for vaccine hesitancy and COVID-19 vaccine rejection by gender, race/ethnicity, family income, and political affiliation. But the direction of association flipped by urbanicity (P=0.0146, with rural dwellers less likely to be COVID-19 vaccine rejecters but more likely to be vaccine hesitant in general), and age (P=0.0037, with fewer pronounced differences across age for COVID-19 vaccine rejection, but a gradient of stronger vaccine hesitancy in general among younger ages). During the COVID-19 epidemic’s early phase, patterns of vaccine hesitancy and COVID-19 vaccine rejection were relatively similar. A significant minority would reject a COVID-19 vaccine, especially one with less-than-ideal effectiveness. Preparations for introducing the COVID-19 vaccine should anticipate substantial hesitation and target concerns, especially among younger adults.

## Introduction

The pandemic of novel coronavirus disease (COVID-19) (1) has caused huge disruptions to life in the United States, which on March 26, 2020, became the country with the most cases globally. By late March 2020, researchers understood the disease to be more severe in older age groups (2), although reports of cases in children and young adults also circulated widely in the news (3).

Widespread uptake of the COVID-19 vaccine could control spread of the disease, but high uptake of vaccine is not guaranteed. Studies during the H1N1 pandemic in 2009 found that many individuals did not want to get vaccinated at the later points during the epidemic (4, 5), which could be due to apathy, desensitization, or a belief that there is a lower probability of illness. Individuals also may be less accepting of a pandemic vaccine if they perceive it to be less safe or effective (6). Because newly developed vaccines have not been on the market long, the general population may perceive these vaccines to be less safe and want more information on the safety profile of the vaccine (7, 8). Additionally, given the proclivity of RNA viruses like SARS-CoV-2 to mutate rapidly, it is not entirely clear how effective any potential vaccine will be. While all vaccines go through rigorous clinical trials (9), members of the general public may not understand this process well. For these reasons, assessing how perceived effectiveness and safety could influence acceptance of a potential COVID vaccine over the course of an outbreak is important. Moreover, the currently available COVID-19 vaccines all have varying attributes in terms of efficacy and risk of adverse events (10).

Vaccine hesitancy, an increasingly recognized global phenomenon (11), could also play a role in limiting people’s desires for a COVID-19 vaccine (12), or could itself be impacted by the epidemic (13). Vaccine hesitancy is defined by the WHO as the “delay in acceptance or refusal of vaccines despite availability of vaccine services. Vaccine hesitancy is complex and context specific, varying across time, place and vaccines. It is influenced by factors such as complacency, convenience and confidence” (14). Over the course of the 2009 H1N1 outbreak, negative attitudes towards vaccination in general in France increased dramatically from 9.6% to 38.2% (15). This could be correlated with decreases in risk perceptions, but more information is needed on how risk perceptions, vaccine hesitancy, and vaccine acceptance interrelate for an emerging outbreak of an infectious disease. Given the rapid development of a COVID-19 vaccine, and its deployment among adults, who have fewer vaccination recommendations than children, it will be important to document how vaccine hesitancy in general differs from the specifics of COVID-19 vaccine rejection.

Another question remains about whether acceptance of a vaccine would vary by age of the individual or safety/effectiveness profile of the vaccine. Anecdotally, it is thought that younger adults are not taking the virus seriously, with frequent news stories about young adults taking spring break trips (16), and news in the early phase of the pandemic focused on risks in older adults. The aims of this study are to estimate differences in vaccine hesitancy and COVID-19 vaccine acceptance by generation, and to characterize if acceptance is affected by how safe or effective the vaccine is.

Understanding vaccination attitudes at the beginning of the epidemic is uniquely important because research from previous epidemics has shown that acceptance of vaccines and compliance towards public health recommendations decline over time (4, 15, 17). Additionally, understanding to what extent US adults would accept a new vaccine for COVID-19 would help the government to design risk communication messages regarding the deployment of new vaccines for COVID-19.

## Methods

### Study Population

US adults who were part of the sampling frame of the survey research firm, Dynata, were eligible for inclusion into this study. Dynata recruits participants through social media and other advertisements, and notifies them of their eligibility to participate in surveys. We built an age-gender nested quota system into the model, whereby a set number of individuals were sought across female/male gender and six age groups (18-24 years old, 25-34 years old, 35-44 years old, 45-54 years old, 55-64 years old, and 65-99 years old), with numbers roughly equivalent to their distribution in the US population. This cross sectional survey was implemented March 20-22, 2020.

We sought a sample size of 800. At this size, with an alpha of 0.05 and a power of 80%, and a proportion of 50% (a statistically conservative estimate of what proportion of the population supports a given public health action) the margin of error is 4%, which we judged to be sufficiently precise.

### Questionnaire

Participants responded to a similar set of questions, but participants who mentioned that they had a parent over the age of 60 or a child under the age of 18 were asked additional questions. The questionnaire is publicly available: https://doi.org/10.6084/m9.figshare.13303121. The questionnaire was pre-tested in 16 individuals ranging in age from early 20s to late 60s.

#### Outcome Variables

The study had two outcomes: potential COVID-19 vaccine rejection and vaccine hesitancy. We asked all participants whether they would accept a hypothetical COVID-19 vaccine. Individuals were randomized into four conditions, where the safety and effectiveness attributes of the COVID-19 vaccine changed. Across the four categories, participants read that the vaccine was either (1): 95% effective with a 5% risk of fever, (2) 50% effective with a 5% risk of fever, (3) 95% effective with a 20% risk of fever, or (4) 50% effective with a 20% risk of fever.

Vaccine hesitancy came from a 10-item scale developed by the World Health Organization (WHO) Strategic Advisory Group of Experts on Immunization (SAGE) Vaccine Hesitancy Working Group (18). Because the original scale’s developers’ original purpose was to assess parental attitudes towards pediatric vaccination, we modified the scale to ask about the individual’s own vaccinations, not their child’s. Participants responded about their agreement on 10 different statements on a 5-point Likert scale. In the analysis, we reordered the responses for certain questions (L1-L4, L6-L8) so that for all items, an increase represented greater vaccine hesitancy. Overall this scale had good internal reliability, the standardized Cronbach alpha was 0.89. The psychometric properties of the original pediatric scale have been previously studied (19). We summed this scale (possible range from 10-50), and then dichotomized the scale at 25, based on a validated measure (20).

#### Independent Variables

The primary independent variable was respondent age, which we categorized by generation. Due to a limited number of responses among individuals of the “Silent Generation” (individuals ≥75 years old) they were collapsed in with Baby Boomers (56-74 years old) for analysis. GenX included individuals 40-55 years old, Millennials 24-39 years old, and GenZ 18-23 years old (21).

For demographics, we used similar wording to previous questionnaires. Participants responded to the same race/ethnicity questions that are on the US Census and the 2019 Behavioral Risk Factor Surveillance System (BRFSS) (22). Due to participant sample sizes, we collapsed the race/ethnicity categories into non-Hispanic White, non-Hispanic Black, Hispanic, and other. We asked about gender identity using guidelines from the American Association of Public Opinion Researchers (23), although no one selected an “other” gender in this survey. A question on urbanicity came from the National Health Interview Survey (24).

We also asked about perceived risk of being infected within the next month using a scale from 0% to 100%. A previous study of H1N1 influenza included a similar question (5). We considered this variable to be continuous in the analysis.

### Statistical Analysis

We ran multivariable logistic regression models, corresponding to the two different outcomes: COVID-19 vaccine rejection and general vaccine hesitancy. We used the same set of demographic predictors (participant gender, urbanicity, generation, race/ethnicity, family income, and political affiliation) based on *a priori* considerations. For vaccine rejection, we also included general vaccine hesitancy, perceived risk of infection, and the safety and effectiveness characteristics as additional independent variables in a “full model”. To assess the interaction of generation and perceived risk, we included a cross-product term between these variables. We calculated the least squares marginal means for each outcome by generation to account for confounding by covariates in the multivariable regression models. We display parameter estimates and 95% confidence intervals (CI).

We compared the strength of odds ratios in the vaccine hesitancy and COVID-19 vaccine rejection by creating two observations per person, with the outcome of one of these observations being for vaccine hesitancy and the other for vaccine rejection. We then specified an interaction term between every predictor variable and a dummy variable for whether this was the hesitancy or vaccine rejection outcome. The model included Generalized Estimating Equation (GEE) methods with an independent correlation matrix to account for two data points per individual. A similar approach was used in a previous study (25). We display the P-value from the interaction terms.

All data were analyzed in SAS version 9.4 (SAS Institute, Cary, NC), and plots were generated in R version 3.6.0 (R Foundation for Statistical Computing, Vienna, Austria).

### Ethical Approval

This study was deemed exempt by the University of Michigan Health Sciences and Behavioral Sciences Institutional Review Board (#HUM00179335). Participants read an information sheet which explained the risks and benefits of the study, which they had to agree to prior to starting the questionnaire. Participants were not given a direct research incentive but were given reward points through Dynata which they could use to exchange for gift cards.

## Results

In total,1,068 individuals clicked on the link to start the online survey and responded to at least one question: 271 (25.4%) did not respond to any questions beyond the screening questions (age and gender) on the start screen, and 50 (4.7%) did not consent, leaving 747 participants (70.0%). We excluded 34 individuals (4.6%) who spent less than 3 minutes on the survey, leaving a total sample size of 713.

**Table 1** shows demographic characteristics of the study population, and the proportion who are vaccine hesitant or who would reject a COVID-19 vaccine by group. The sample was demographically diverse. Study participants were 54.3% female and 32.5% said they lived in a rural area. A plurality, about one-third (34.1%), were ≥56 years old, a majority (74.5%) were non-Hispanic White, and most participants reported family income either between $2,000-$4,999 (28.5%) or $5,000-$9,999 (30.5%).

**Table 1:** Demographics of online survey panel, United States, March 2020.

		Count (column %)	Vaccine hesitant (row %)	Reject COVID-19 vaccine (row %)
Overall		713 (100%)	230 (33.0%)	131 (18.4%)
Participant’s gender	Male	326 (45.7%)	98 (31.0%)	51 (15.6%)
	Female	387 (54.3%)	132 (34.8%)	80 (20.7%)
Participant’s residence	Rural	227 (32.5%)	88 (40.2%)	37 (16.3%)
	Urban	471 (67.5%)	139 (29.9%)	93 (19.7%)
Participant’s generation	Baby boomer and silent generation	242 (34.1%)	48 (20.5%)	40 (16.5%)
	GenX	222 (31.3%)	60 (27.6%)	41 (18.5%)
	Millennial	176 (24.8%)	80 (46.2%)	32 (18.2%)
	GenZ	70 (9.9%)	41 (59.4%)	17 (24.3%)
Participant’s race/ethnicity	Non-Hispanic White	531 (74.5%)	146 (28.0%)	86 (16.2%)
	Non-Hispanic Black	50 (7.0%)	33 (70.2%)	17 (34.0%)
	Hispanic	53 (7.4%)	24 (47.1%)	12 (22.6%)
	Other	79 (11.1%)	27 (36.0%)	16 (20.3%)
Monthly family income	<$2,000	140 (20.2%)	70 (51.1%)	39 (27.9%)
	$2,000-$4,999	198 (28.5%)	70 (36.3%)	43 (21.7%)
	$5,000-$9,999	212 (30.5%)	60 (28.7%)	30 (14.2%)
	≥$10,000	144 (20.7%)	27 (19.1%)	18 (12.5%)
Political affiliation	Republican	216 (31.8%)	71 (33.3%)	37 (17.1%)
	Democrat	262 (38.5%)	76 (29.7%)	41 (15.6%)
	Independent	202 (29.7%)	74 (37.6%)	51 (25.2%)
Perceived risk of infection within next month	*median (IQR)*	*32% (11%-51%)*	–	–

### COVID-19 Vaccine Rejection

**Figure 1:**
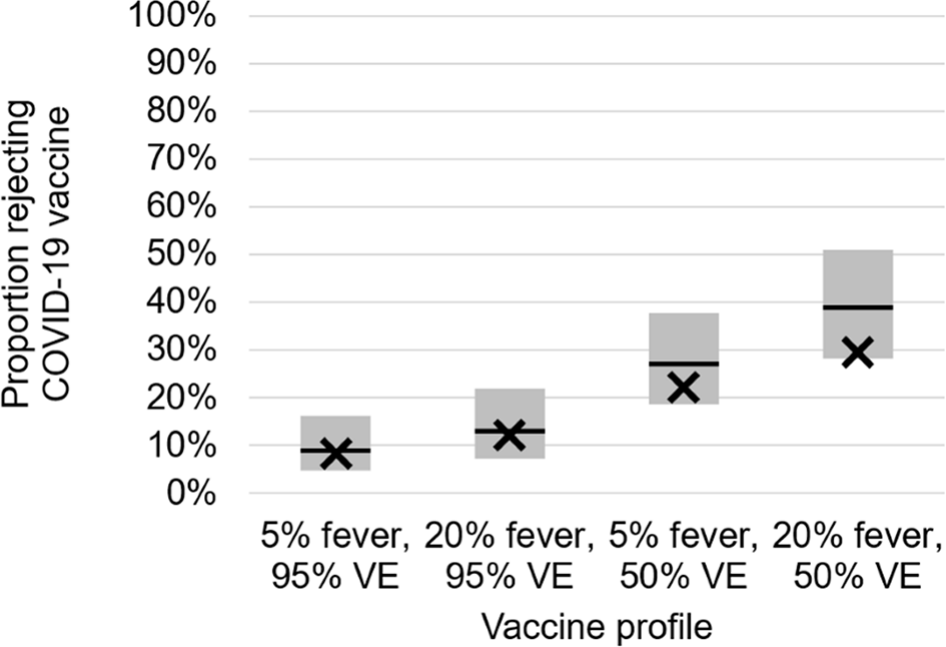
Modeled (bars) and observed values (X) for vaccine rejection by vaccine effectiveness (VE) and risk of fever. Modeled estimates and 95% confidence intervals from least square means marginal proportions, accounting for age, urbanicity, race/ethnicity, income, and political affiliation.

Overall, 8.4% of individuals would reject a hypothetical COVID-19 vaccine that was 95% effective with a 5% risk of fever, whereas 12.2% would for a vaccine that was 95% effective and had a 20% risk of fever, 22.2% would for a vaccine 50% effective with a 5% risk of fever, and 29.5% would for a vaccine 50% effective with a 20% risk of fever (**Figure 1**). In the multivariable model for vaccine rejection accounting for vaccine attributes, vaccine hesitancy, risk perceptions, and the interaction between generation and risk perceptions (**Table 2**), we found that all these variables were significant. A vaccine with a 20% risk of fever had 1.63 times greater odds of being rejected compared to a vaccine with only a 5% risk (95% CI: 1.03, 2.57), and a vaccine 50% effective had 4.08 times greater odds of being rejected compared to a vaccine with a 95% effectiveness (95% CI: 2.44, 6.83). These differences translate to 95% effective vaccines being rejected by 12.8% of the population (95% CI: 8.6%, 18.7%), whereas 50% effective vaccines were rejected by 33.0% (95% CI: 25.6%, 41.4%). There was a smaller disparity by safety: a vaccine with a 5% risk of fever would be rejected by 17.5% (95% CI: 12.5%, 23.9%) and this was 25.5% (95% CI: 18.8%, 33.7%) for a vaccine with a 20% risk of fever.

**Table 2:** Impact of demographic factors on general vaccine hesitancy and COVID-19 vaccine rejection, online survey panel, US, March 2020.

	COVID-19 vaccine rejection (full model) OR (95% CI)	COVID-19 vaccine rejection (abbreviated model) OR (95% CI)	Vaccine hesitant OR (95% CI)	P-value^a^
Participant’s gender				0.3494
Male	ref	ref	ref	
Female	1.34 (0.82, 2.18)	1.36 (0.90, 2.06)	1.09 (0.76, 1.56)	
Participant’s residence				0.0146
Rural	0.61 (0.36, 1.03)	0.74 (0.48, 1.16)	1.36 (0.93, 1.97)	
Urban	ref	ref	ref	
Participant’s generation				0.0037
Baby Boomer (≥56 years)	0.54 (0.19, 1.50)	1.11 (0.63, 1.94)	0.40 (0.25, 0.65)	
GenX (40-55 years)	0.81 (0.31, 2.10)	1.16 (0.67, 1.99)	0.54 (0.34, 0.85)	
Millennial (24-39 years)	ref	ref	ref	
GenZ (18-23 years)	1.20 (0.35, 4.16)	1.19 (0.58, 2.45)	1.34 (0.71, 2.51)	
Participant’s race/ethnicity				0.7793
Non-Hispanic White	ref	ref	ref	
Non-Hispanic Black	1.87 (0.80, 4.39)	2.86 (1.40, 5.87)	4.07 (1.96, 8.42)	
Hispanic	1.29 (0.54, 3.07)	1.44 (0.69, 3.03)	1.56 (0.81, 2.99)	
Other	2.76 (1.25, 6.10)	1.76 (0.89, 3.49)	1.35 (0.72, 2.53)	
Monthly family income				0.5541
<$2,000	0.91 (0.49, 1.69)	1.25 (0.74, 2.11)	1.62 (1.00, 2.63)	
$2,000-$4,999	ref	ref	ref	
$5,000-$9,999	0.59 (0.32, 1.08)	0.60 (0.35, 1.03)	0.76 (0.48, 1.20)	
≥$10,000	0.68 (0.33, 1.39)	0.53 (0.29, 1.00)	0.44 (0.25, 0.77)	
Political affiliation				0.4363
Republican	0.78 (0.43, 1.41)	0.77 (0.47, 1.27)	1.10 (0.70, 1.71)	
Democrat	0.71 (0.41, 1.26)	0.48 (0.29, 0.78)	0.58 (0.37, 0.90)	
Independent	ref	ref	ref	
Vaccine hesitant				
No	ref	–	–	
Yes	5.56 (3.39, 9.11)	–	–	
Increase in 1 percentage point in perceived risk	0.97 (0.95, 0.98)	–	–	
Vaccine safety				
5% fever risk	ref	–	–	
20% fever risk	1.63 (1.03, 2.57)	–	–	
Vaccine effectiveness				
95% effective	ref	–	–	
50% effective	4.08 (2.44, 6.83)	–	–	
Generation * perceived risk interaction				
Risk * Baby Boomer	1.03 (1.01, 1.06)	–	–	
Risk * GenX	1.02 (1.00, 1.05)	–	–	
Risk * GenZ	0.99 (0.96, 1.03)	–	–	

a Difference in estimates from COVID-19 vaccine rejection model and vaccine hesitancy model.

**Figure 2:**
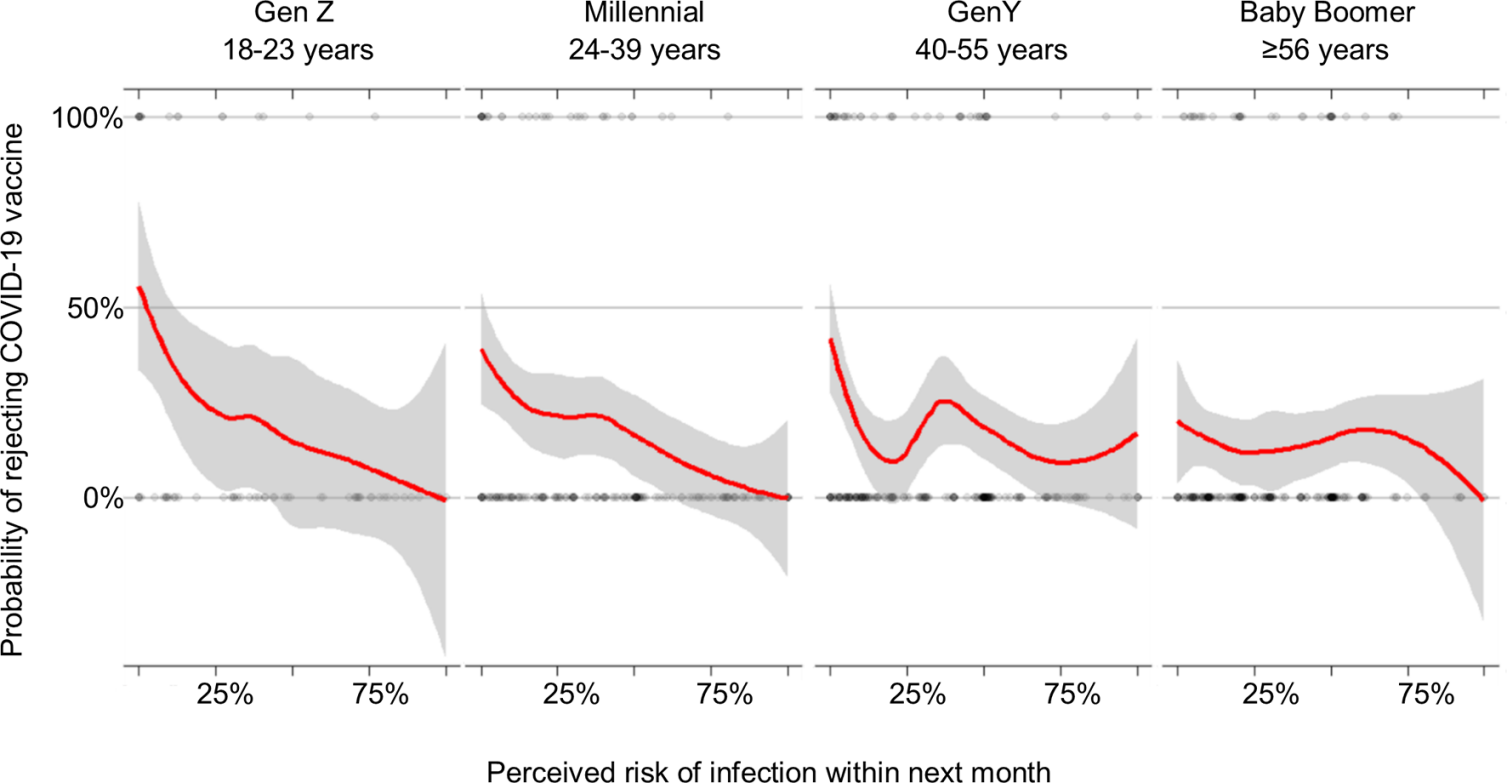
Relation between risk perceptions and COVID-19 vaccine acceptance, by generation, US, March 2020.

Vaccine hesitancy and perceived risk were significantly associated with COVID-19 vaccine rejection. Those vaccine hesitant were significantly more likely to reject COVID-19 vaccination (OR: 5.56, 95% CI: 3.39, 9.11). Increases in risk perceptions were associated with decreases in vaccine rejection (OR: 0.97, 95% CI: 0.95, 0.98). The association of risk perceptions and vaccine rejection varied by generation, with significant attenuation for Baby Boomers versus Millennials. **Figure 2** shows how the slope of the relationship between risk perceptions and vaccine acceptance is sharper for later generations: for Baby Boomers there is less of a relationship between risk perception and vaccine acceptance, whereas this is highly apparent for GenZ.

### Comparison of COVID-19 Vaccine Rejection and General Vaccine Hesitancy

**Table 2** shows results from multivariable models for COVID-19 vaccine rejection and vaccine hesitancy using the same set of predictors. There was no significant difference in COVID-19 vaccine rejection by generation, however there was a significant generational difference in vaccine hesitancy. Baby Boomers (OR: 0.40, 95% CI: 0.25, 0.65) and GenX (OR: 0.54, 95% CI: 0.34, 0.85) had lower odds of vaccine hesitancy compared to Millennials. The difference in the strength of association between generation and vaccine hesitancy and between generation and vaccine rejection was significant (P=0.0037).

Race/ethnicity was significantly related to both COVID-19 vaccine rejection and vaccine hesitancy, and the strengths of association between race/ethnicity and both outcomes were similar. COVID-19 vaccine rejection was higher in non-Hispanic Black individuals compared to non-Hispanic White individuals (OR: 2.86, 95% CI: 1.40, 5.87). And we found that participants who were non-Hispanic Black also had higher levels of hesitancy (OR: 4.07, 95% CI: 1.96, 8.42) than participants non-Hispanic White.

Higher levels of income were associated with less COVID-19 rejection and lower vaccine hesitancy scores. The association between income and COVID-19 rejection and between income and vaccine hesitancy was similar. For example, vaccine rejection was lower in those with higher income (>$10,000 *vs* $2,000-$4,999 OR: 0.53, 95% CI: 0.29, 1.00), and for this same comparison the odds of vaccine hesitancy was 0.44 (95% CI: 0.25, 0.77).

Political affiliation was related to vaccine rejection and vaccine hesitancy. Those identifying as Democrats were less likely to reject the COVID-19 vaccine and less likely to be vaccine hesitant compared to Independents.

## Discussion

This study examines acceptance of a COVID-19 vaccine, and how it is affected by vaccine hesitancy in the early phase of the COVID-19 epidemic. We surveyed a demographically diverse group of U.S. adults between March 20 and 22, 2020. During this interval the estimated number of cases increased from 18,747 to 33,404. Our study found generational differences in vaccine hesitancy, with less hesitancy in older adults. However, this did not translate into reduced acceptance of the COVID-19 vaccine among younger adults.

In our study, a large majority of individuals would accept a COVID-19 vaccine, but a small and significant minority stated they would reject it. As expected, US adults were more accepting of a COVID-19 vaccines if they were safer or more effective. We do not know how safe or effective the COVID-19 vaccine will be, but if it mimics the influenza vaccine (26), it could be similar to our profile of 50% effectiveness and 5% risk of fever, which would be rejected by almost one-fourth of the population. Because we found differences in vaccine rejection by race/ethnicity and income, there could also be spatial differences in vaccine rejection, and therefore pockets of susceptibility within the country.

COVID-19 vaccine acceptance may also change over time. Two previous cross-sectional surveys this year found that between late January and late February 2020, acceptance of a COVID-19 vaccine increased from 48% to 65% (27). As the outbreak becomes more real to Americans, their acceptance of a vaccine may increase. This finding, in turn, would relate to the positive relationship we found between risk perceptions and vaccine acceptance, which has been echoed in other research (28). It is worthwhile for future research to observe the changes of vaccine acceptance and how it is related to the spread of disease and actions taken by the government.

Vaccine hesitancy may also increase over the course of the COVID-19 pandemic. In a study in France during the 2009 H1N1 influenza outbreak, negative attitudes towards vaccination increased rapidly, with the researchers speculating this was correlated both with concerns about the safety of a newly introduced H1N1 influenza vaccine and with heightened controversy over the perceived seriousness of the vaccine (15).

If vaccine hesitancy does increase, this could differentially impact younger generations and lead to lower uptake among younger adults. Therefore, how we deliver effective messages to the groups with high vaccine hesitancy to influence their behaviors is critical. A study of adult preferences for vaccines found that provider recommendations were just as important as effectiveness of the vaccine (8). Accordingly, strong promotion from health professionals could counter lower effectiveness of the vaccine.

We found that the relationship between risk perceptions and vaccine acceptancy varies by generation. One of the possible explanations could be that older generations are highly accepting of vaccines, regardless of their risk perceptions, whereas younger generations have higher intent when they perceive their personal risk to be higher. Future research could explain the reasons for this discrepancy, but it could be possibly tied to experience with previous outbreaks/pandemics, more appreciation for vaccines across the life-span, or more experience with vaccine-preventable diseases, such as measles, polio, or pertussis, which are now relatively rare. Regardless, vaccine education among younger generations should also focus on increasing risk perceptions. These promotions will be important for two reasons. One, if perceived risk decreases over time, as it has in previous outbreaks (4, 5), younger adults may become even more less likely to be vaccinated. Two, similar to the influenza vaccine (26), the COVID-19 could be even less effective in older adults compared to younger adults. Maintaining high vaccination coverage in younger adults could be key to creating adequate herd immunity that protects older adults.

General vaccine hesitancy itself was strongly related to rejection of the COVID-19 vaccine. There is already concern in some anti-vaccine groups that a COVID-19 vaccine could be compulsory (29). Our study found that vaccine hesitancy was higher in individuals among those with lower monthly incomes. This finding contrasts with previous research which has found that those with higher income tend to have higher vaccine hesitancy, lower vaccine coverage (30, 31), and higher incidence of vaccine-preventable disease (32). However, other studies have found no such relationship (33, 34). In contrast to many previous studies focusing on parents’ hesitancy to pediatric vaccines, our study asked adult participants about their hesitancy to adult vaccination. It is likely that patterns of vaccine hesitancy differ when directed at an adult rather than at their children. For example, a previous study which presented participants with information about influenza vaccines with different attributes found that parents were more risk sensitive when considering vaccinating their child than considering the vaccines for themselves (35). And another study which looked separately at preferences among parents for childhood vaccines and adults for adult vaccines found that effectiveness was more important in the analysis of parents than in the analysis of adults (8).

### Strengths and limitations

This survey used Internet-based samples to allow rapid data collection during the pandemic and to avoid person-to-person contact. However, Internet samples may have inherent biases. There is sampling bias in that individuals who participate need to have access to the internet, and so individuals of lower socioeconomic status will be less likely to participate. Additionally, individuals may answer rapidly with little thought, which is why we removed individuals from our analytical sample who completed the survey in a short period of time. We also note that constructs in our study, including items related to vaccine hesitancy or interpretations of effectiveness or fever, could differ across participants. Other factors, like education, could impact vaccination behaviors, but were not included in the survey.

## Conclusions

In this survey of US adults in late March 2020, we found that a large majority of individuals would accept a COVID-19 vaccine. However, about one-third would reject the vaccine if it was only 50% effective – which is a reasonable estimate compared to the seasonal influenza vaccine. In general we found similar patterns for vaccine hesitancy and COVID-19 vaccine rejection, indicating that thoughts about vaccinations in general and for COVID-19, specifically, are highly correlated. Vaccine hesitancy may increase over the course of the outbreak, and if vaccine hesitancy increases and perceived risk of infection decreases, younger adults in particular may be less likely to become vaccinated. Acknowledging generational differences in risk perceptions could help the government tailor messages to promote vaccines. Additionally, stressing the safety of the vaccine will be important when rolling out the COVID-19 vaccine.

## Data Availability Statement

The datasets presented in this study can be found in online repositories. The names of the repository/repositories and accession number(s) can be found below: https://doi.org/10.3886/E130422V1.

## Ethics Statement

This study was reviewed by the University of Michigan Institutional Review Board (#HUM00176454). Before starting the survey, participants read an informed consent form, which they could download as a PDF, and clicked a button to agree.

## Author Contributions

S-FS conceptualized the study and wrote the original draft. AW obtained funding, conceptualized the study, contributed to visualization, and wrote the first draft. NM wrote the original draft, and contributed to visualization. LP and BZ-F contributed to methodology, and contributed critically to reviewing the manuscript. YL conceptualized the study and contributed critically to reviewing the manuscript. All authors contributed to the article and approved the submitted version.

## Funding

This study was supported by the National Institute of Allergy and Infectious Diseases of the National Institutes of Health under Award Number K01AI137123 and by an award from the National Science Foundation, Division of Social and Economic Sciences (#2027836).

## Conflict of Interest

The authors declare that the research was conducted in the absence of any commercial or financial relationships that could be construed as a potential conflict of interest.
